# Healthcare resource use and costs related to surgical infections of tibial fractures in a Spanish cohort

**DOI:** 10.1371/journal.pone.0277482

**Published:** 2022-11-11

**Authors:** Mariano Barrés-Carsí, Jorge Navarrete-Dualde, Javier Quintana Plaza, Elena Escalona, Christian Muehlendyck, Thibaut Galvain, José Baeza, Antonio Balfagón

**Affiliations:** 1 Trauma Unit, Unit of Orthopaedic Surgery and Traumatology, Hospital Universitario y Politécnico La Fe de Valencia, Valencia, Spain; 2 Johnson & Johnson Medical, Health Economics and Market Access, Madrid, Spain; 3 Johnson & Johnson Medical GmbH, Health Economics and Market Access, Norderstedt, Germany; 4 Johnson & Johnson Medical, Health Economics and Market Access, Issy les Moulineaux, France; 5 Sepsis Unit, Unit of Orthopaedic Surgery and Traumatology, Hospital Universitario y Politécnico La Fe de Valencia, Valencia, Spain; Assiut University Faculty of Medicine, EGYPT

## Abstract

Surgical site infection constitutes a serious complication in the healing process of bone fractures and has been associated with increases in medical resource use and healthcare costs. This study evaluates the economic impact of surgical site infection in tibial fractures in a Spanish cohort. It is a retrospective, single-centre, comparative cohort study of patients with tibial fractures with longitudinal follow-up for up to 18 months post-surgery. Included patients (n = 325) were adults, with tibial fracture, either isolated or polyfracture, or polytrauma with an Injury Severity Score >15. Patients had been surgically treated within 30 days of the tibial fracture by external or internal fixation, or external followed by internal fixation. Most patients (84.9%) had an American Society of Anaesthesiology score of 1–2. 20% of the patients had one open tibial fracture, 12.3% had polytrauma, and 20% had multiple fractures. Most patients were treated with a nail (41.8%) or a plate (33.8%). 56 patients (17.2%) developed surgical site infection. Patients with infection had significantly higher hospital length of stay (34.9 vs 12.0 days; p<0.001; +191%), readmissions (1.21 vs 0.25; p<0.001; +380%) and mean operating theatre time (499 vs 219 min; p<0.001; +128%) than patients without infection. Mean length of stay in intensive care did not significantly increase with infection (2.8 vs 1.7 days; p = 0.25). Total in-hospital costs for patients with infection increased from €7,607 to €17,538 (p<0.001; +131%). Overall, infections were associated with significantly increased healthcare resource use and costs. Preventive strategies to avoid infections could lead to substantial cost savings.

## Introduction

Surgical site infection (SSI) is a concerning complication in orthopaedic and trauma surgery due to its potentially devastating consequences for patients such as reoperation, readmission, delayed recovery, transient or permanent loss of function in the affected region and death [1–3]. Tibial fractures are particularly prone to this complication. SSI may lead to prolonged treatment, compromised clinical outcomes and, in some cases, limb amputations [4, 5]. Moreover, more than 15% of tibial fractures are open, representing the most common open fracture site for long bones [6]. In open fractures, the Gustilo-Anderson (GA) classification is prognostically useful for predicting orthopaedic infections [7]. In tibial fractures, despite the use of systemic antibiotics, patients may experience superficial or deep infection with an overall rate of about 1–4% [8, 9]. Deep infection rates range from 1% after operative fixation of closed low-energy fractures, to up to 30% in complex open fractures [10, 11].

SSI has been associated with an increase in medical resource use and treatment costs [12–17]. Deep infections—those which invade muscle, fascia, or bone—in particular, pose a significant economic burden to healthcare systems. In tibial shaft fractures, for instance, the infection after internal fixation significantly increases in-hospital costs, length of in-hospital stay (LOS), readmissions, and reoperations associated with tibial fracture fixation [18].

In the Spanish setting, there is a dearth of data that quantify the economic burden of SSI after fixation of tibial fractures. From an European perspective, in a Danish study of patients with open tibial fractures treated with a free flap, the presence of an infection increased mean LOS from 28 to 63.8 days, and mean treatment costs from €49,301 to €67,958 [19]. A study in Belgium revealed treatment costs to be approximately 6.5 times higher in patients with SSI compared to those without infection [20]. Among British patients with tibial osteomyelitis, the LOS for those treated with limb salvage procedures was 15 days (range 19–27 days), and treatment cost was €16,718; for patients who required limb amputation, LOS was 13 days (range 8–17 days), and treatment cost was €18,441 [21]. In Spain, the economic burden of SSI in patients with tibial fractures is not well documented, and no scientific literature was identified to provide information about the treatment pathways or the costs of care. This retrospective study aims to evaluate the economic burden associated with SSI on in-hospital healthcare resource use (iHHRU) and associated costs, in patients surgically treated for tibial fracture in a Spanish hospital.

## Methods

### Study design

This is a retrospective cohort (level of evidence III) study of patients from the Trauma Unit of the “Hospital Universitario y Politécnico La Fe” between January 1^st^, 2010 to August 31^st^, 2017. This Unit is specialised in trauma care, infection prevention and treatment. Patients were followed-up for a 18-month period after the hospital admission for a tibial fracture (index date). The exposure was the presence of SSI at the tibial fracture site within 18 months of hospital admission. Clinical data were collected from electronic medical records by hospital staff researchers. Hospital costs were estimated based on healthcare resources consumption (registered in patients records) and the associated tariffs of these resources (**S1 Table**). The tariffs (publicly available) are representing payments made to hospitals from national healthcare public system to cover hospital costs. Hospital costs considered were implant costs, operating room costs, laboratory costs, hospital stay costs, intensive care costs, imaging costs and other costs.

Infections were classified according to clinical practice in the Trauma Unit of the Hospital Universitario y Politécnico La Fe. They were identified as deep or superficial based on the surgeon’s diagnosis and the results of the sample wound cultures. Deep infections were classified in the presence of two positive wound cultures, whereas superficial infections had two negative wound cultures.

We obtained ethical approval from the Ethical Committee of “Hospital Universitario y Politécnico La Fe”. The study was considered exempt from patients’ informed consent due to its retrospective design.

### Inclusion and exclusion criteria of study participants

Included patients were 18 years and older and hospitalized with a closed or open tibial fracture between 2010 to 2017, with an Injury Severity Score (ISS) >15, and external or internal fixation within 30-days of fracture. Patients were excluded if they had pathological fracture (e.g., due to a tumour) or a hospitalization for pre-existing chronic disorders or co-morbidities unrelated to trauma care. They were also excluded if they had active infection at the site prior to surgical fixation treatment or if the hospitalisation was for a revision procedure (defined as previous treatment with an internal or external fixation within the last 6 weeks before transfer to the hospital of the study). Polytrauma patients with an Abbreviated Injury Scale (AIS) score ≥3 [22–24] in more than four body regions and patients with bilateral tibial fracture were excluded. Patients with AIS ≥3 in more than four body regions [24] were excluded from the study to minimize the bias for patients whose treatment costs were primarily driven by injuries other than the tibial fracture.

### Study data

General characteristics of patients were extracted from medical records: age, sex, year of admission to hospital, weight, American Society of Anesthesiologists (ASA) score [25], diabetes (Yes/No), immunosuppressed (Yes/No), and Arbeitsgemeinschaft für Osteosynthesefragen Foundation/Orthopaedic Trauma Association (AO/OTA) classification [26]. The independent variable was the presence of SSI (Yes/No) at the tibial fracture site within 18 months of hospital admission. The primary outcome was total in-hospital costs over 18 months (continuous variable). Secondary outcomes consisted of the following iHHRUs (continuous variables unless specified otherwise): (i) hospital stay related including LOS in the hospital general ward, LOS in the intensive care unit (ICU) and the number of hospital admissions and readmissions; (ii) surgery related including the operating theatre (OT) time, debridement procedure (Yes/No), osteotomy (Yes/No), drains, wound vacuum (Yes/No), pressure irrigation (Yes/No), flap reconstruction (Yes/No), skin graft (Yes/No), implant removal (Yes/No), external fixation removal (Yes/No), bone grafting with bone morphogenetic protein (BMP) (Yes/No) and bone grafting with other materials (Yes/No); and (iii) non-surgery related care including laboratory tests and imaging exams. Secondary outcomes also included the costs associated with the iHHRUs (continuous variables) and consisted of LOS costs in general ward and in ICU, implant costs, operating theatre costs (staff and non-staff related), laboratory and imaging costs and other costs.

### Statistical analysis

Frequency counts and proportions were calculated for binary and categorical variables. Mean, median, quartiles and standard deviation (SD) were calculated for continuous variables. Bivariate comparisons were made by infection status (Yes/No) for baseline characteristics and economic outcomes according to their statistical distribution. Normal distribution was assessed (i) based on summary statistics, (ii) graphically using histograms and qqplots and (iii) conducting Shapiro Wilk, Lilliefors and Kolmogorov Smirnov tests. Multivariate methods were constructed to examine outcomes after adjustment for sex, age, ASA score, open/closed fracture and polytrauma. We used multivariate logistic regression models for categorical outcomes (Yes/No). Generalized linear models [GLM] with a gamma distribution and log link were used for continuous costs outcomes as they were not normally distributed, right skewed, and strictly positive [27]. Count outcomes were adjusted using GLM with either a Poisson distribution and a log link or a Gamma distribution and log link. Zero-inflated negative binomial regressions were utilized for ICU outcomes considering overdispersion and excess zeros as not all the patients were admitted in ICU. The following subgroup analyses were conducted for the primary outcome: (i) superficial vs. deep infection, (ii) open vs. closed fracture, (iii) polytrauma vs. isolated trauma, and (iv) polyfracture vs. isolated fracture. The study was powered to detect differences in the primary outcome, statistical significance was set a-priori at p<0.05 (one-sided).

## Results

### Baseline characteristics

The cohort included 325 patients among whom 56 patients (17.2%) developed an infection (**Table 1**). Of those 40 (71.4%) were deep infections (**S3 Table**). In the infection group there were more males (78.6% vs 58.7%) (p<0.008) and more patients with an open fracture vs. a closed fracture (58.9% vs 11.9%) (p<0.001) (**Table 1**). SSI was more frequent in higher-degree fractures, in polytrauma patients and in patients with multiple fractures. There were no statistical differences in age group, ASA score, or immunosuppressed and diabetes status, between the groups with and without SSI (more data in **S2 Table**).

**Table 1:** Baseline characteristics of all the patients and with or without SSI of tibial fracture.

	All patients	Patients without SSI	Patients with SSI	p-value
	N = 325	N = 269	N = 56	
**Sex, N (%)**				
Male	202 (62.2)	158 (58.7)	44 (78.6)	0.008
**Age (years), N (%)**			** **	
18 to 35	88 (27.1)	76 (28.3)	12 (21.4)	
36 to 55	125 (38.5)	98 (36.4)	27 (48.2)	0.44
56 to 75	87 (26.8)	74 (27.5)	13 (23.2)	
76 or more	25 (7.7)	21 (7.8)	4 (7.1)	
**ASA score, N (%)**		** **	** **	
1	128 (39.4)	113 (42.0)	15 (26.8)	
2	148 (45.5)	116 (43.1)	32 (57.1)	0.09
3 or more	49 (15.1)	40 (14.9)	9 (16.1)	
**Diabetes, N (%)**				
Yes	14 (4.3)	11 (4.1)	3 (5.4)	0.71
**Immunosuppressed patient, N (%)**	** **	** **	** **	
Yes	11 (3.4)	7 (2.6)	4 (7.1)	0.10
**Open fracture, N (%)**	** **	** **	** **	
Yes	65 (20.0)	32 (11.9)	33 (58.9)	<0.001
**Fracture degree, N (%)**	** **	** **	** **	
0	260 (80.0)	237 (88.1)	23 (41.1)	
I	23 (7.1)	17 (6.3)	6 (10.7)	
II	24 (7.4)	12 (4.5)	12 (21.4)	<0.001
III*	2 (0.6)	0	2 (3.6)	
IIIA	11 (3.4)	3 (1.1)	8 (14.3)	
IIIB	3 (0.9)	0	3 (5.4)	
IIIC	2 (0.6)	0	2 (3.6)	
**Polytrauma, N (%)**				
Yes	40 (12.3)	22 (8.2)	18 (32.1)	<0.001
**Polyfracture, N (%)**	** **	** **	** **	
Yes	65 (20.0)	42 (15.6)	23 (41.1)	<0.001
**Treatment, N (%)**	** **	** **	** **	
ExFix	24 (7.4)	11 (4.1)	13 (23.2)	
ExFix+nail	11 (3.4)	6 (2.2)	5 (8.9)	
ExFix+plate	44 (13.5)	24 (8.9)	20 (35.7)	<0.001
nail	136 (41.8)	128 (47.6)	8 (14.3)	
plate	110 (33.8)	100 (37.2)	10 (17.9)	

*Sub-type of degree III not available; ASA score: American Society of Anesthesiologists Classification; SSI: surgical site infection.

### Primary outcome

The unadjusted mean (SD) in-hospital cost at 18 months was €26,289 (€20,588) for patients with SSI vs. €8,061 (€9,742) for patients without SSI. In the multivariate analysis (GLM), in-hospital costs at 18 months were 131% [95% CI: 85%; 188%] higher for patients with SSI than for those without SSI (p<0.001). Adjusted mean in-hospital costs at 18 months for patients with infection were €17,538 (95% CI: €14,397; €21,364) vs. €7,607 (95% CI; €7,012; €8,252) (p<0.001) (**Table 2**).

**Table 2:** Bivariate and multivariate results for the differences in costs (at 18 months) between patients without and with SSI of tibial fracture.

Variable		Bivariate analysis		Multivariate analysis*	
	SSI	Mean (SD) (€)	p-value	Mean [95% CI] (€)	p-value
*Primary*	No	8,061 (9,742)	p < 0.001	7,607 [7,012; 8,252]	p < 0.001
Total in-hospital costs	Yes	26,289 (20,588)		17,538 [14,397; 21,364]	
*Secondary-Cost component*					
		
LOS in general ward costs	No	1,781 (2,520)	p < 0.001	1,629 [1,483; 1,790]	p < 0.001
	Yes	6,601 (4,572)		4,746 [3,777; 5,964]	
LOS in ICU costs	No	1,649 (7,193)	p < 0.001	2,361 [1,419; 3,304]	NS
	Yes	8,458 (13,757)		3,791 [1,852; 5,730]	
Implant costs	No	1,196 (825)	p < 0.001	1,182 [1,081; 1,292]	p < 0.001
	Yes	2,043 (1,854)		2,114 [1,703; 2,624]	
Non-staff-related OT costs	No	2,581 (1,343)	p < 0.001	2,627 [2,448; 2,820]	p < 0.001
	Yes	7,108 (7,089)		5,983 [5,041; 7,101]	
Staff-related OT costs	No	400 (208)	p < 0.001	407 [379; 437]	p < 0.001
	Yes	1,102 (1,099)		927 [781; 1,101]	
Imaging costs	No	762 (286)	p < 0.001	775 [725; 828]	0.003
	Yes	1,149 (563)		1,030 [872; 1,216]	
Lab costs	No	149 (251)	p < 0.001	115 [66; 199]	0.001
	Yes	363 (273)		322 [252; 411]	
Other costs	No	44 (110)	NS	43 [30; 56]	NS
	Yes	70 (142)		76 [31; 121]	

*Multivariate analysis after adjustment for sex, age, ASA score, open/closed fracture and polytrauma. CI: confidence interval; ICU: intensive care unit; LOS: length of stay; NS: non-significant difference; OT: operating theatre; SD: standard deviation; SSI: surgical site infection.

### In-hospital healthcare resource use

**Fig 1:**
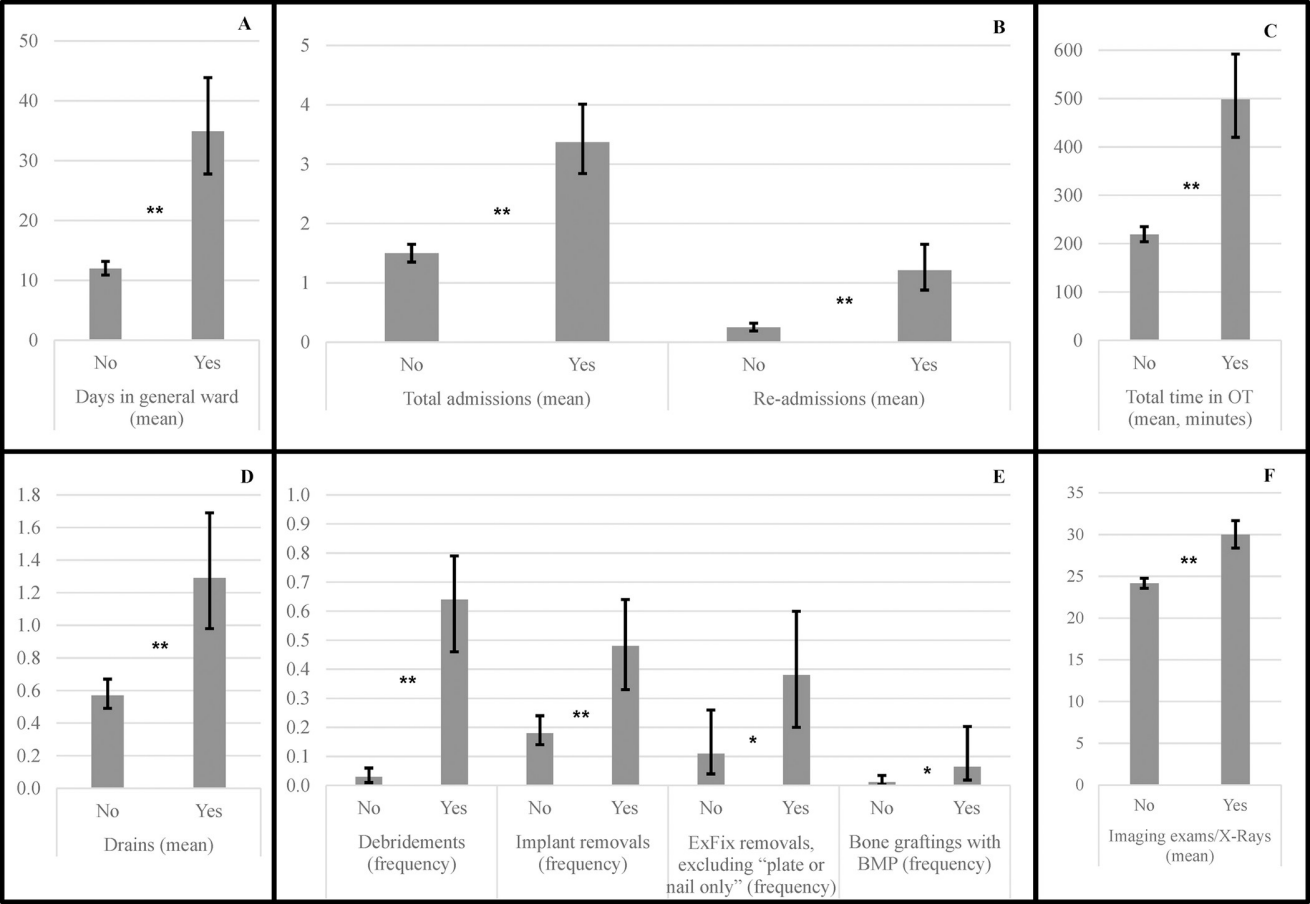
Differences in iHHRU at 18 months after tibial fracture with/without SSI (multivariate analysis). A, B, C, D, E and F show the components of iHHRU with significant differences between patients with SSI (Yes) and without SSI (No) in the multivariate analysis. ExFix: external fixation; BMP: bone morphogenetic protein; OT: operating theatre. Error bars represent the 95% confidence intervals. *p<0.05; **p<0.001.

In the multivariate analysis of general iHHRUs and associated costs over 18 months, mean LOS was 34.9 days for patients with SSI vs. 12.0 days (p<0.001) for patients without SSI (difference of 191% [95% CI: 125%; 277%]). Mean OT time was 128% (95% CI: 88%; 176%) higher, at 499 minutes for those with SSI vs. 219 minutes for those without SSI (p<0.001). Patients with SSI had overall greater mean [95% CI] number of admissions (1.5 [1.4; 1.7] vs. 3.4 [2.8; 4.0]; +126% [83%; 178%]; p<0.001), readmissions (+386%; p<0.001), debridements (Odds Ratio (OR) = 63.6; p<0.001), implant removals (OR = 4.2; p<0.001), external fixation removal (10.6% vs. 37.8%; p = 0.027), mean number of drains (0.57 vs. 1.29; p<0.001), bone grafting with BMP (OR = 6.0; p = 0.047), and imaging exams (+24%; p<0.001) compared to those without SSI (**Fig 1**). Mean, cumulative ICU days were 1.7 days vs. 2.8 days for patients with infection; results were not statistically significant (p = 0.25). There was no statistical difference in osteotomy (OR = 6.1; p = 0.2) or in bone grafting with other materials (OR = 1.3; p = 0.6). The following were not included within in the multivariate model given that they were present only among SSI patients: 2 amputations (p = 0.002), 10 vacuum-assisted closures (p<0.001), 6 pressure irrigations (p<0.001), 14 flap reconstructions (p<0.001), and 16 skin grafts (p<0.001).

### In-hospital cost

In the multivariate analysis, several cost components were significantly higher for patients with SSI: LOS in general ward, staff-related OT, non-staff-related OT, implant, imaging, and laboratory costs. Patients with SSI had overall greater mean general ward costs (+191% [95% CI: 125%; 277%]; p<0.001), implant costs (+79% [95% CI: 40%; 128%]; p<0.001), imaging costs (+33% [95% CI: 10%; 60%]; p = 0.003), OT costs (staff- and non-staff-related) (+128% [95% CI: 88%; 176%]; p<0.001), and laboratory costs (+180% [95% CI: 51%; 420%]; p<0.001). ICU costs were not statistically significantly higher in patients with SSI after adjustment (p = 0.24) (**Table 2**).

### Subgroup analyses

#### Deep vs. superficial infection

Patients with deep infection incurred mean total 18-month unadjusted costs of €26,906 vs. €24,747 for patients with superficial SSI. Deep infection costs were not significantly different from those of superficial infection in bivariate analysis (p = 0.64) or after adjustment (p = 0.9). In bivariate analysis, superficial or deep infection were associated with higher mean costs than for patients with no infection (€8,061; p<0.001).

#### Opened/closed tibial fracture

There were greater rates of infection in open fracture (50.8%) than closed fracture (8.9%). The mean unadjusted cost of patients with infection and open fracture was €33,319, while it was €16,204 in patients with closed fracture. Infection increased costs in both groups (p<0.001). After adjustment, the increased costs from infection in patients with open fractures were significantly higher than in those with closed fractures (€23,234 vs €14,033; p = 0.047).

#### Polytrauma vs. isolated trauma

In patients with polytrauma, the mean unadjusted cost with infection was €38,923, compared to €27,606 without infection (p = 0.038). Whereas, the mean cost of patients with infection without polytrauma was €20,305, compared to €6,320 in patients without infection nor polytrauma (p<0.001).

#### Polyfracture vs. isolated fracture

In patients with polyfracture, the mean cost with infection was €36,351, compared to €19,392 without infection (p<0.001). Whereas, the mean cost of patients with isolated fracture and with infection was €19,276, compared to €5,965 in patients with isolated fracture and without infection (p<0.001) (**S3 Table**).

## Discussion

This study confirms the finding of previous studies [12–17] that general iHHRUs and associated costs were higher in cases where an SSI was present than in cases where it was not present. The multivariate analysis demonstrated total in-hospital costs at 18 months for patients with SSI increased by 131% (from €7,607 to €17,538; p<0.001). Non-staff-related OT costs (from €2,627 to €5,983), costs of LOS in general ward (from €1,629 to €4,746), imaging (from €775 to €1,030), staff-related OT (from €407 to €927) and laboratory costs (from €115 to €322) were all significantly higher in patients with SSI. Moreover, there was an increase in the cost of implants used among SSI patients (from €1,182 to €2,114), due to a greater number of implant removals (nails and plates), and a greater need for external fixator to better control the healing of the infected soft tissue. Regarding the use of resources, patients with SSI had significantly greater LOS (+191%), number of readmissions (+386%), and OT time (+128%) than patients without SSI.

In a study of 358 patients with fracture fixation of the tibia, treatment costs were approximately 6.5 times higher in patients with infection than in those without infection [20]. In a study of patients with severe open tibial fractures treated with a free flap, the cost of treatment was 62% higher in patients with infected than uninfected fractures [19]. In our study an increase of in-hospital costs for patients with SSI by 131% was seen for the whole study group covering open and closed fractures, various types of tibial fracture treatments, and diverse degrees of severity. In England, a recent retrospective study of patients with and without infection following intramedullary nailing for a tibial shaft fracture has shown significant increases in in-hospital costs (80%), LOS (109% increase at 1 year), readmissions (5.18 times at 1 year), and reoperations associated with infection (2.47 times at 1 year) [18].

In our study, with a heterogenous group including tibial diaphysis, proximal and distal tibia, polytrauma, polyfractures, etc., 17.2% of the 325 patients had SSI. Most of the patients with Gustilo-Anderson (GA) III experienced an infection (15/18). This study substantiates previous findings showing that the more severe the fracture, the higher the risk of infection [28]. In order to reflect routine clinical practice and minimize variability, it was decided to include polytrauma patients (12.3%) in our study with specific inclusion criteria (ISS >15), even though their inclusion poses certain methodological challenges; such as ensuring consistency in the definition of polytrauma patients, and considering the inherent heterogeneity of this patient population. It was observed that SSI was more frequent in polytrauma patients (45%) than in non-polytrauma patients (13.3%); and there were higher costs in polytrauma patients with SSI (€38,923) than in polytrauma patients without SSI (€27,606).

Post-surgery infections of tibial fractures involve significant increases in inpatient costs, LOS, readmissions, and reoperations [18]. Thus, treatments that prevent the development of SSI are necessary. In a retrospective study of patients with open tibia fracture, Metsemakers et al. [29], found that no deep infection occurred after placement of a gentamicin-coated nail. A meta-analysis found that patients with severe fractures had materially lower infection rates when they received locally delivered antibiotics prophylactically that those receiving standard systemic antibiotics [30]. An economic model of the potential cost-effectiveness of the use of antibiotic-coated intramedullary nails in open tibial fractures indicates that infection rates, inpatient days, reoperations, and cost reduction may be associated with the use of antibiotic-coated implants. And so, infection and reoperation rates could thereby potentially be reduced by 72% and 10% respectively, saving up to 15% of hospital costs in patients at high risk of infection [31]. Clinical recommendations are still lacking for routine use of antibiotic-loaded implant pending more robust evidence on efficacy and also on safety related to resistance development.

Currently, there is a debate in the scientific community about the concept of superficial and deep SSI. The Centers for Disease Control and Prevention (CDC) consider that SSI can be superficial or deep [32]. Metsemakers et al. [33] have stated that it is not possible to distinguish between these two kinds of infections in specified fractures such as tibial fractures, and therefore considered them as infections of the fracture site. Recently, the AO Foundation and the European Bone and Joint Infection Society have proposed a consensus definition for fracture-related infection (FRI) to standardise the diagnostic criteria and improve the quality of patient care [34]. This consensus group considered the following clinical local signs as suggestive of FRI: local redness, swelling, increase of local temperature, fever (≥38.3°C), or persistent, increasing or new onset wound drainage beyond the first few days after surgery. These suggestive criteria are not pathognomonic and should therefore prompt the treating surgeon to further investigate the possibility of an FRI. In our study, the infection was considered as deep or superficial based on the surgeon’s diagnosis of positive clinical signs (pus, open wound), and their subsequent wound-culture results. Neither the CDC guidelines nor Metsemakers et al.’s recommendations were followed, because: 1) data gathering started in 2010 in order to fulfil our sample size criteria, and not all variables were available at that time; 2) the distinction between deep and superficial was retained as such in order to follow clinical practice definitions from the Hospital Universitario y Politécnico La Fe; and 3) tibial SSI, as stated by Metsemaker et al., is a controversial infection site, since only a few millimetres separate fascia from bone and there is no surrounding soft tissue.

The present study had certain limitations, firstly this was a retrospective cohort study without randomization. The study collected limited patient demographics and patient co-morbidities, and so the study could not adjust for unknown and unmeasured confounders potentially leading to bias [35]. This was also a single-centre study, which limits the generalizability of the results. In addition, the diagnosis of infection was not based on CDC guidelines nor on Metsmakers et al.’s recommendations. The study was also only powered for the primary endpoint. In terms of the economic assessment, it was limited to the in-hospital setting, as it was not possible to follow-up patients outside the hospital potentially underestimating the total economic burden of infection throughout the entire care pathway. Antibiotic therapy costs were also excluded, and so the economic burden of infection might have been underestimated. Lastly, external cost reference data were used, and indirect costs such as productivity losses were not considered.

## Conclusions

Adult patients with a diagnosis of tibial fracture (isolated or polytrauma/polyfracture) and surgically treated within 30 days in this retrospective cohort study showed that, over 18 months, infections were associated with significantly increased healthcare resource use and associated costs. Preventive strategies to avoid infections could lead to substantial cost savings for the Spanish healthcare system.

## Supporting information

S1 Table References of costs. (DOCX)

S2 Table Tibial fracture type according to the fracture and dislocation classification compendium 2018. *Meinberg E, Agel J, Roberts C, Karam M, Kellam J. Fracture and Dislocation Classification Compendium—2018. International Comprehensive Classification of Fractures and Dislocations Committee. J Orthop Trauma [Internet]. 2018 [cited 2020 May 15];32:S1–10. Available from: http://journals.lww.com/00005131-201801001-00001.
(DOCX)

S3 Table Differences in total costs (at 18 months) between patients without and with SSI of tibial fracture, by sub-groups. *Multivariate analysis after adjustment. SD: standard deviation; SE: standard error; SSI: surgical site infection.
(DOCX)
