# Supplementary material for: Healthcare resource use and costs related to surgical infections of tibial fractures in a Spanish cohort
Source: PLoS One. 2022 Nov 11;17(11):e0277482. doi: 10.1371/journal.pone.0277482 (PMC9651570; doi:10.1371/journal.pone.0277482)
Supplement: S1 Table — (DOCX) [file pone.0277482.s001.docx]

| **Cost item** | **Unit cost value (€)** | **Reference** |
| --- | --- | --- |
| Nail | 660 | Internal ASP averaged over these categories: non-sterile steel, sterile steel, non-sterile titanium, sterile titanium |
| Plate | 961 | Internal ASP averaged over these categories: non-sterile steel, sterile steel, non-sterile titanium, sterile titanium |
| External Fixator (ExFix) | 490 | Internal ASP averaged over these categories: non-sterile steel, sterile steel, non-sterile titanium, sterile titanium |
| Additional surgery | 11,338.85 | Government DRG MESO1 - Postoperative, post-traumatic and other device infections with surgical procedure (moderate severity) |
| Additional reconstructive surgery | 17,976.35 | Average of 2 government DRG MESO1 (moderate degree + severe degree) - 312 Diagnosis of skin graft due to musculoskeletal and connective tissue disorders, except hand |
| Vacuum- assisted closure | 95.69 | Norway reference from 2006 updated to 2018 10.1080/17453670610013051 |
| Cost per day in general ward | 136 | *Presidencia de la Generalitat* *(Valencia) (2018) Ley 20/2017, de 28 de Diciembre, de tasas. Boletin Oficial del Estado*  *Nº 38 12 de Febrero 2018* |
| Cost per day in ICU | 1365 | *Presidencia de la Generalitat* *(Valencia) (2018) Ley 20/2017, de 28 de Diciembre, de tasas. Boletin Oficial del Estado*  *Nº 38 12 de Febrero 2018* |
| X-ray | 25.31 | *Presidencia de la Generalitat* *(Valencia) (2018) Ley 20/2017, de 28 de Diciembre, de tasas. Boletin Oficial del Estado*  *Nº 38 12 de Febrero 2018* |
| Computed Tomography (CT) | 147.51 | *Presidencia de la Generalitat* *(Valencia) (2018) Ley 20/2017, de 28 de Diciembre, de tasas. Boletin Oficial del Estado*  *Nº 38 12 de Febrero 2018* |
| Magnetic Resonance Imaging (MRI) | 280 | *Presidencia de la Generalitat* *(Valencia) (2018) Ley 20/2017, de 28 de Diciembre, de tasas. Boletin Oficial del Estado*  *Nº 38 12 de Febrero 2018* |
| Blood culture | 28.24 | *Presidencia de la Generalitat* *(Valencia) (2018) Ley 20/2017, de 28 de Diciembre, de tasas. Boletin Oficial del Estado*  *Nº 38 12 de Febrero 2018* |
| Biochemistry tests | 10.75 | *Presidencia de la Generalitat* *(Valencia) (2018) Ley 20/2017, de 28 de Diciembre, de tasas. Boletin Oficial del Estado*  *Nº 38 12 de Febrero 2018* |
| Coagulation tests | 3.54 | *Presidencia de la Generalitat* *(Valencia) (2018) Ley 20/2017, de 28 de Diciembre, de tasas. Boletin Oficial del Estado*  *Nº 38 12 de Febrero 2018* |
| Complete Blood Count (CBC) | 3.54 | *Presidencia de la Generalitat* *(Valencia) (2018) Ley 20/2017, de 28 de Diciembre, de tasas. Boletin Oficial del Estado*  *Nº 38 12 de Febrero 2018* |
| Arterial blood gas test | 11 | *Presidencia de la Generalitat* *(Valencia) (2018) Ley 20/2017, de 28 de Diciembre, de tasas. Boletin Oficial del Estado*  *Nº 38 12 de Febrero 2018* |
| Operating theatre non-staff related per min | 12 | Hospital Procedures Catalogue. *Comunidad Valenciana 2005* |
| Operating theatre scrub nurse per min | 0.22 | Hospital Procedures Catalogue. *Comunidad Valenciana 2005* |
| Operating theatre surgeon per min | 0.4 | Hospital Procedures Catalogue. *Comunidad Valenciana 2005* |
| Operating theatre nurse per min | 0.22 | Hospital Procedures Catalogue. *Comunidad Valenciana 2005* |
| Operating theatre anaesthesiologist per min | 0.4 | Hospital Procedures Catalogue. *Comunidad Valenciana 2005* |
| Reamer/Irrigator/Aspirator (RIA) | 1,092 | Data from DePuy Synthes Spain |
| Bone grafting Bone Morphogenetic Protein (BMP) | 2,920 | Data from DePuy Synthes Spain |
| Bone grafting other material | 1,500 | Data from DePuy Synthes Spain |
| Blood extraction per min | 0.22 | Hospital Procedures Catalogue. *Comunidad Valenciana 2005* |
