# Supplementary material for: Healthcare resource use and costs related to surgical infections of tibial fractures in a Spanish cohort
Source: PLoS One. 2022 Nov 11;17(11):e0277482. doi: 10.1371/journal.pone.0277482 (PMC9651570; doi:10.1371/journal.pone.0277482)
Supplement: S2 Table — *Meinberg E, Agel J, Roberts C, Karam M, Kellam J. Fracture and Dislocation Classification Compendium—2018. International Comprehensive Classification of Fractures and Dislocations Committee. J Orthop Trauma [Internet]. 2018 [cited 2020 May 15];32:S1–10. Available from: http://journals.lww.com/00005131-201801001-00001. (DOCX) [file pone.0277482.s002.docx]

| **AO/OTA classification***  **N (%)** | **All patients**  **N=325** | **Patients without SSI** | **Patients with SSI** |
| --- | --- | --- | --- |
| 41A1  41A2  41A3  41A3+42A1  41B1  41B2  41B3  41B3+42C3  41C1  41C2  41C3  42A1  42A2  42A3  42B1  42B2  42B3  42C1  42C2  42C3  43A1  43A2  43A3  43B1  43B2  43B3  43C1  43C2  43C3 | 4 (1.23)  6 (1.85)  6 (1.85)  1 (0.31)  14 (4.31)  6 (1.85)  32 (9.85)  1 (0.31)  11 (3.38)  9 (2.77)  7 (2.15)  46 (14.2)  31 (9.54)  15 (4.62)  14 (4.31)  15 (4.62)  4 (1.23)  3 (0.92)  5 (1.54)  4 (1.23)  20 (6.15)  6 (1.85)  17 (5.23)  1 (0.31)  10 (3.08)  1 (0.31)  8 (2.46)  11 (3.38)  17 (5.23) | 1 (0.37)  6 (2.23)  4 (1.49)  0  13 (4.83)  5 (1.86)  30 (11.2)  0  9 (3.35)  5 (1.86)  3 (1.12)  43 (16.0)  28 (10.4)  14 (5.20)  14 (5.20)  12 (4.46)  4 (1.49)  2 (0.74)  4 (1.49)  3 (1.12)  17 (6.32)  5 (1.86)  13 (4.83)  1 (0.37)  9 (3.35)  1 (0.37)  6 (2.23)  7 (2.60)  10 (3.72) | 3 (5.36)  0  2 (3.57)  1 (1.79)  1 (1.79)  1 (1.79)  2 (3.57)  1 (1.79)  2 (3.57)  4 (7.14)  4 (7.14)  3 (5.36)  3 (5.36)  1 (1.79)  0  3 (5.36)  0  1 (1.79)  1 (1.79)  1 (1.79)  3 (5.36)  1 (1.79)  4 (7.14)  0  1 (1.79)  0  2 (3.57)  4 (7.14)  7 (12.5) |
