# Supplementary material for: Healthcare resource use and costs related to surgical infections of tibial fractures in a Spanish cohort
Source: PLoS One. 2022 Nov 11;17(11):e0277482. doi: 10.1371/journal.pone.0277482 (PMC9651570; doi:10.1371/journal.pone.0277482)
Supplement: S3 Table — *Multivariate analysis after adjustment. SD: standard deviation; SE: standard error; SSI: surgical site infection. (DOCX) [file pone.0277482.s003.docx]

| **Subgroups** |  | **Bivariate analysis** | **Multivariate analysis*** | |
| --- | --- | --- | --- | --- |
|  | **SSI (N)** | **Mean (SD) (€)** | | **Mean (SE) (€)** |
| Closed tibial fracture | No (237) | 8,068 (10,171) | | 7,397 (318) |
|  | Yes (23) | 16,204 (9,566) | | 14,033 (1,952) |
| Open tibial fracture | No (32) | 8,009 (5,733) | | 7,979 (926) |
|  | Yes (33) | 33,319 (23,270) | | 23,324 (2,770) |
| No Infection  Deep infection  Superficial infection | No (269) | 8,061 (9,742) | | 7,607 (316) |
|  | Yes (40)  Yes (16) | 26,906 (20,626)  24,747 (21,083) | | 17,611 (2,035)  17,348 (3,028) |
| Polytrauma  Isolated trauma | No (22) | 27,606 (24,065) | | 24,025 (3,377) |
|  | Yes (18) | 38,923 (19,517) | | 29,607 (4,752) |
|  | No (247) | 6,320 (4,093) | | 6,543 (272) |
|  | Yes (38) | 20,305 (18,455) | | 17,658 (1,949) |
| Polyfracture | No (42) | 19,392 (20,334) | | 16,559 (1,514) |
|  | Yes (23) | 36,351 (23,925) | | 29,182 (3,813) |
| Isolated fracture | No (227) | 5,965 (3,043) | | 6,235 (248) |
|  | Yes (33) | 19,276 (14,542) | | 16,849 (1,823) |
